# The Alpha-defensin Test for Periprosthetic Joint Infection Responds to a Wide Spectrum of Organisms

**DOI:** 10.1007/s11999-015-4152-x

**Published:** 2015-01-29

**Authors:** Carl Deirmengian, Keith Kardos, Patrick Kilmartin, Simmi Gulati, Patrick Citrano, Robert E. Booth

**Affiliations:** 1 CD Diagnostics Inc, Claymont, DE USA; 2 Lankenau Institute for Medical Research, The Rothman Institute, Thomas Jefferson University, 100 Lancaster Avenue, MOB 456, Wynnewood, PA 19096 USA; 3 Aria 3B Orthopaedic Specialists, Philadelphia, PA USA

## Abstract

**Background:**

The alpha-defensin test has been previously demonstrated to be highly accurate in the diagnosis of prosthetic joint infection (PJI), nearly matching the Musculoskeletal Infection Society definition for PJI. However, the relationship between alpha-defensin levels and differing infecting organism has not yet been investigated.

**Questions/purposes:**

The purpose of this study is to describe the breadth of organisms that can trigger a positive synovial fluid alpha-defensin test result in the setting of PJI and also to assess the magnitude of the alpha-defensin result in terms of various pathogen characteristics.

**Methods:**

Between December 2012 and March 2014, one laboratory processed 2319 synovial fluid samples for alpha-defensin testing. The present study reviewed the results of the 1937 samples that simultaneously had a synovial fluid culture performed; these came from 418 surgeons in 42 states. The overall culture-positive rate was 49% (244 of 498) among alpha-defensin-positive synovial fluids and 1% (19 of 1439) among alpha-defensin-negative synovial fluids. The organisms recovered from 244 alpha-defensin-positive, culture-positive fluids were recorded and grouped based on various characteristics, including Gram type, species, virulence, oral pathogenicity, and source joint. Alpha-defensin-negative samples served as uninfected controls. Median alpha-defensin levels were calculated for each group, and Dunn’s multiple comparison test for nonparametric data was used to identify any statistically significant (p < 0.05) organism-specific differences in the alpha-defensin level.

**Results:**

The alpha-defensin test for PJI was positive in the setting of a wide spectrum of organisms typically causing PJI. The median alpha-defensin level for all 244 alpha-defensin-positive, culture-positive samples (4.7 [interquartile range {IQR}, 3.7–5.3]) was higher than negative controls (0.26 [IQR, 0.22–0.33]) with a median difference of 4.4 (p < 0.001). There were no differences in the median alpha-defensin levels when performing a multiple comparison test among Gram-positive organisms (4.7 [IQR, 3.6–5.3]), Gram-negative organisms (4.8 [IQR, 4.2–5.3]), yeast (4.1 [IQR, 2.2–5.1]), virulent organisms (4.7 [IQR, 3.8–5.2]), less virulent organisms (4.8 [IQR, 3.6–5.4]), oral pathogens (4.5 [IQR, 3.2–5.2]), knees (4.7 [IQR, 3.7–5.3]), hips (4.9 [IQR, 4.1–5.8]), or shoulders (5.3 [IQR, 4.0–10.7]) with all comparisons having a p > 0.999.

**Conclusions:**

The alpha-defensin test provides consistent results regardless of the organism type, Gram type, species, or virulence of the organism and should be seriously considered to be a standard diagnostic tool in the evaluation for PJI. Future research should focus on the performance of this test in specific clinical scenarios such as the immediate postoperative period in the setting of severe immunocompromise and in the setting of a native joint.

**Level of Evidence:**

Level III, diagnostic study.

## Introduction

The synovial fluid alpha-defensin test is an immunoassay that was specifically developed, through genomic and proteomic studies, to aid in the diagnosis of prosthetic joint infection (PJI). In several studies, the alpha-defensin test has been shown to closely match the final postoperative result of the complex criteria-based Musculoskeletal Infection Society (MSIS) definition for PJI [2, 5–7], which was initially developed to standardize clinical diagnosis [16] and was subsequently modified by consensus [11]. Additionally, the alpha-defensin test has been demonstrated to outperform the leukocyte esterase reagent test, which is used by some to diagnose PJI [5]. The simplicity of the alpha-defensin test, combined with its ability to provide a clinical result soon after a preoperative joint aspiration, makes it an attractive tool for diagnosing PJI.

The alpha-defensin protein is an antimicrobial peptide that is naturally released by neutrophils responding to a pathogen in the synovial fluid [9]. It is therefore very reasonable to question whether this antimicrobial peptide is released in response to all pathogens and also whether the alpha-defensin test can detect PJIs caused by all organisms. The alpha-defensin test is a qualitative immunoassay optimized and validated for synovial fluid [6], providing the alpha-defensin concentration in terms of a signal-to-cutoff ratio (S/CO). Although alpha-defensin test results are clinically reported as “positive” or “negative”, the S/CO value reflecting the concentration of alpha-defensin in each synovial fluid sample is available for research purposes.

The purpose of this study is to describe the breadth of organisms that can trigger a positive synovial fluid alpha-defensin test result in the setting of PJI and also to assess the magnitude of the alpha-defensin result in terms of various pathogen characteristics. Specifically, we sought to determine whether a broad range of organisms and organism characteristics, including of Gram staining characteristics, specific species, virulence, oral or nonoral, and anatomic source, will trigger differential alpha-defensin levels.

## Patients and Methods

This study was approved by the institutional review board.

The Synovasure^®^ Test (Citrano Laboratories, subsidiary of CD Diagnostics, Towson, MD, USA) includes a qualitative synovial fluid immunoassay for human alpha-defensin(1–3). The test is indicated to aid in the diagnosis of PJI. We retrospectively evaluated deidentified laboratory data for the purposes of this study. Between December 2012 and March 2014, one laboratory (Citrano Laboratories in Towson, MD, USA) processed 2319 synovial fluid samples for alpha-defensin testing. The present study reviewed the results of the 1937 samples, which simultaneously had a synovial fluid culture performed at the same laboratory. Therefore, all data analyzed in this study were generated at one central laboratory. All 1937 synovial fluid samples were specifically sent to the laboratory in the setting of a workup of PJI and came from 418 surgeons in 42 states. These synovial fluid samples were mailed overnight in plastic laboratory tubes and specific biohazard packaging, and there were no documented episodes of contamination or tube compromise. The methods of sample handling, transportation, and alpha-defensin testing in this study were identical to that of a previous study, which demonstrated an alpha-defensin sensitivity and specificity of 100% and 95%, respectively, for the diagnosis of PJI [2].

The alpha-defensin test results and synovial fluid cultures evaluated for this study were generated in one laboratory for the purpose of providing a clinical result for a requesting physician. An alpha-defensin test S/CO > 1 is considered a positive result, indicating PJI [6]. The synovial fluid culture results evaluated for this study were generated by BacT/ALERT^®^ FAN FA/FN culture bottles for recovery of both aerobic and anaerobic organisms (Biomerieux, Durham, NC, USA). The organisms were identified and evaluated for susceptibilities using the VITEK^®^ 2 ID/AST system (Biomerieux), a fully automated system that provides rapid microbial identification and susceptibility testing. For synovial fluid sample submissions that specified a shoulder source, culture results in a supplemented broth, to allow for *Proprionibacterium acnes* growth, were available when requested by the physician and were held for 2 weeks.

We selected synovial fluid samples that had both a positive alpha-defensin test for PJI and a positive culture result because the primary aim was to describe the breadth of organisms that can trigger a positive alpha-defensin test result in the setting of PJI. Of 1937 synovial fluid samples, 498 had a positive alpha-defensin test result and 1439 had a negative alpha-defensin test result. The median alpha-defensin negative result was used as a control by which to compare the various groupings of alpha-defensin-positive samples.

Of 498 alpha-defensin positive samples, 244 samples also had a positive culture. We grouped these samples by organism type (Gram-positive, Gram-negative, yeast), species, characteristics (virulent, less virulent, oral, nonoral), and anatomic source (knee, hip, shoulder). Species were only analyzed as a group when there were seven or more samples available. For the alpha-defensin-positive and alpha-defensin-negative sample groups, the proportion of positive synovial fluid culture was calculated. Additionally, the organisms identified were classified by morphology, species, Gram type, and virulence. Organisms classified subjectively as virulent included *Staphylococcus aureus,* *Staphylococcus lugdunensis, Streptococcus agalactiae*, the *Enterobacteriaceae,* and the nonfermenting Gram-negative bacilli. All other organisms were classified as less virulent. Oral pathogens were also subgrouped for analysis, including *Abiotrophia defectiva, Actinomyces meyeri, Granulicatella adiacens, Lactobacillus gasseri, Parvimonas micra, Peptostreptococcus asaccharolyticus, Streptococcus cristatus, Streptococcus gordonii, Streptococcus mitis/oralis, Streptococcus mutans, Streptococcus parasanguinis, Streptococcus sanguinis*, and *Veillonella* species.

When available, the joint aspirated for collection of synovial fluid was noted. Among 244 alpha-defensin-positive, culture-positive samples, surgeons noted the source joint as the knee (N = 173 [71%]), hip (N = 40 [16%]), or shoulder (N = 6 [3%]). The joint was not specified by the requesting surgeon in the remaining 25 submitted fluid samples, and these were excluded from analysis of joint-specific alpha-defensin results but included in all other analyses.

Median alpha-defensin S/CO levels were calculated for alpha-defensin-positive and -negative groups as well as for the various pathogen classifications and anatomic sites. The median alpha-defensin results, when organisms were grouped by various characteristics, were evaluated for statistically significant differences using Dunn’s test for the multiple comparison of nonparametric data. A threshold p value of < 0.05 was used for this analysis, which was adjusted for multiple comparisons. The Mann-Whitney test was used to assess the difference between two nonparametric groups. A threshold p value of < 0.05 was used for this analysis.

**Fig. 1:**
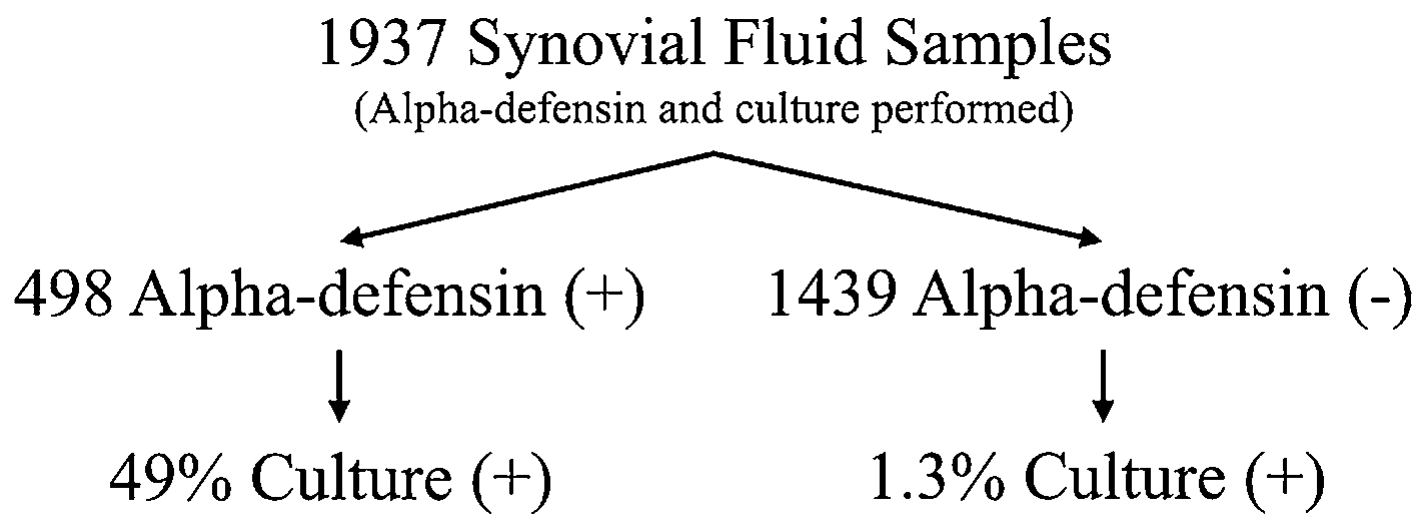
Synovial fluid samples, from patients undergoing arthroplasty, having both an alpha-defensin test and synovial fluid culture, were included in this study. The figure depicts the overall breakdown of results.

Of the 498 alpha-defensin-positive synovial fluid samples, 244 (49%) were also culture-positive. Of 1439 alpha-defensin-negative samples, only 19 (1%) were culture-positive (Fig. 1). A wide spectrum of organisms was isolated from the 244 synovial fluid samples that were positive for both alpha-defensin and culture (Table 1). Gram-positive bacteria alone, Gram-negative bacteria alone, and yeast alone were isolated from 210 (86%), 23 (9%), and six (3%) of these 244 samples, respectively. Five cultures (2%) had polymicrobial growth. Among the 244 culture-positive synovial fluid samples, 77 (30%) resulted in growth of an organism classified as virulent, 167 (70%) resulted in growth of an organism classified as less virulent, and 24 (10%) resulted in growth of an organism classified as an oral pathogen.

**Table 1:** Organisms isolated from alpha-defensin-positive synovial fluid

Organism isolated	Number
*Abiotrophia defectiva*	1
*Achromobacter xylosoxidans*	1
*Actinomyces meyeri*	1
*Bacteroides fragilis*	3
*Burkholderia cepacia group*	2
*Candida albicans*	3
*Candida parapsilosis*	2
*Citrobacter koseri*	1
*Corynebacterium jeikeium*	1
*Corynebacterium striatum*	7
*Enterobacter cloacae complex*	4
*Enterococcus faecalis*	12
*Enterococcus faecium*	2
*Escherichia coli*	2
*Granulicatella adiacens*	2
*Kocuria kristinae*	5
*Kocuria rosea*	1
*Kodamaea ohmeri*	1
*Lactobacillus gasseri*	1
Multiorganism	5
*Parvimonas micra*	3
*Peptostreptococcus asaccharolyticus*	1
*Propionibacterium acnes*	2
*Proteus mirabilis*	3
*Pseudomonas aeruginosa*	5
*Serratia marcescens*	1
*Staphylococcus aureus*	33
*Staphylococcus caprae*	15
*Staphylococcus epidermidis*	78
*Staphylococcus hominis*	1
*Staphylococcus lugdunensis*	14
*Staphylococcus simulans*	2
*Staphylococcus warneri*	3
*Streptococcus agalactiae* (Group B)	11
*Streptococcus cristatus*	1
*Streptococcus gordonii*	1
*Streptococcus mitis/oralis*	7
*Streptococcus mutans*	3
*Streptococcus parasanguinis*	1
*Streptococcus sanguinis*	1
*Veillonella* species	1
Total	244

## Results

The alpha-defensin test for PJI is triggered by infections caused by a wide spectrum of organisms, and there was no difference in the magnitude of the alpha-defensin level, regardless of Gram stain characteristics, specific organism, virulence, oral or nonoral pathogen, or anatomic source.

**Fig. 2:**
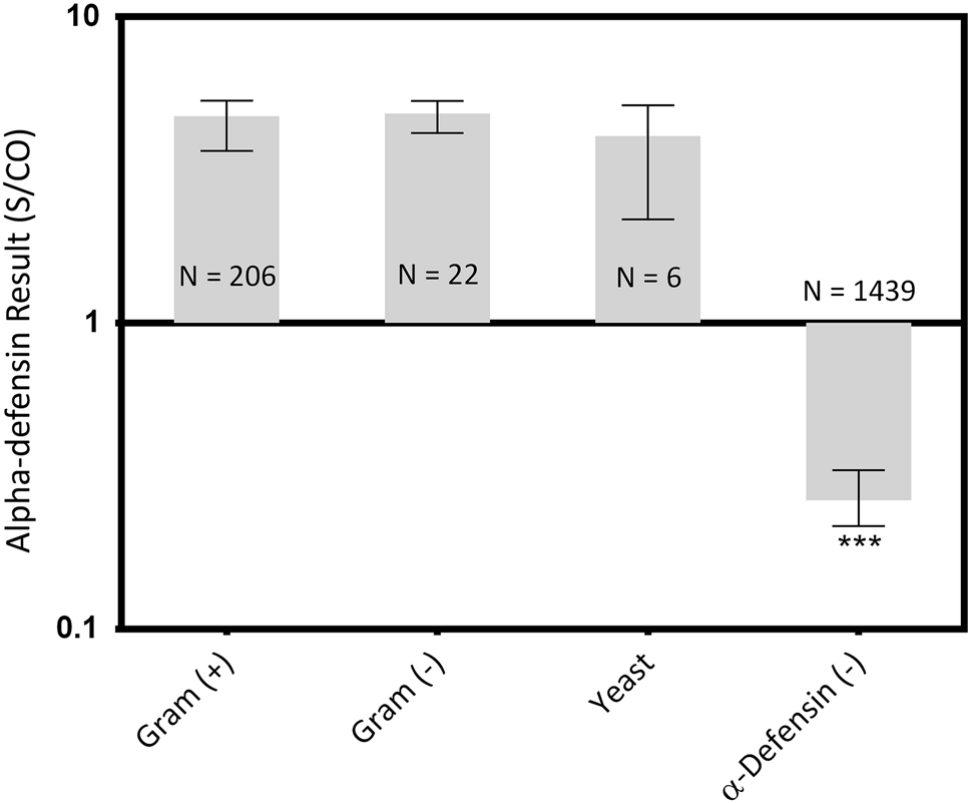
Synovial fluid samples with both a positive alpha-defensin result and a positive culture were grouped by organism type. Median values and the interquartile ranges are plotted on a log scale. *** = Different with statistical significance.

With regard to Gram stain characteristics, all alpha-defensin-positive groups were different than the negative control (Gram-positive median: 4.7 [interquartile range {IQR}, 3.6–5.3], Gram-negative median: 4.8 [IQR, 4.2–5.3], yeast median: 4.1 [IQR, 2.2–5.1]; Gram-positive versus control median difference: 4.4, p < 0.001; Gram-negative versus control median difference: 4.5, p < 0.001; yeast versus control median difference: 3.8, p < 0.001; Fig. 2), whereas no differences in S/CO levels were found between the organism group medians (all p ≥ 0.999).

**Fig. 3:**
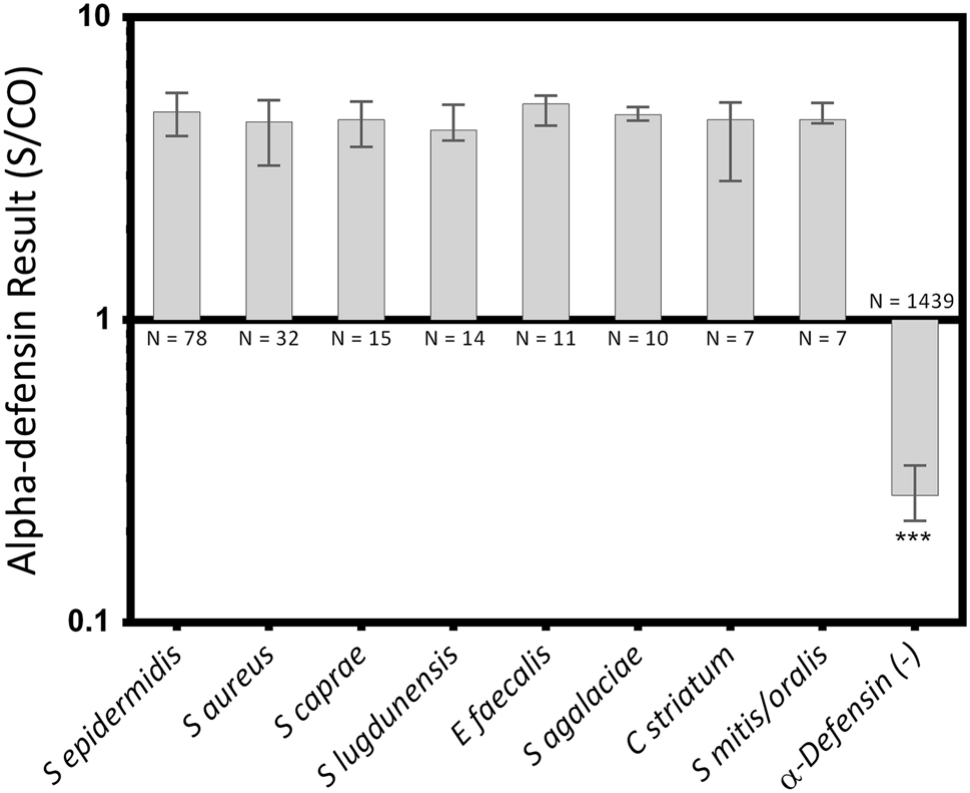
Synovial fluid samples with both a positive alpha-defensin result and a positive culture were grouped by organism species. Median values and the IQRs are plotted on a log scale. *** = Different with statistical significance.

Similarly, when comparing S/CO levels for causative organisms against control levels, all groups were greater than the negative control (*S epidermidis* median 4.9 [IQR, 4.1–5.6], *S aureus* median 4.5 [IQR, 3.2–5.3], *S caprae* median 4.6 [IQR, 3.7–5.3], *S lugdunensis* median 4.2 [IQR, 3.9–5.2], *E faecalis* median 5.2 [IQR, 4.4–5.5], *S agalaciae* median 4.8 [IQR, 4.6–5.1], *C striatum* median 4.6 [IQR, 2.9–5.2], *S mitis/oralis* median 4.6 [IQR, 4.5–5.2]; *S epidermidis* versus control median difference: 4.6, p < 0.001; *S aureus* versus control median difference: 4.3, p < 0.001; *S caprae* versus control median difference: 4.3, p < 0.001; *S lugdunensis* versus control median difference: 4.0, p < 0.001; *E faecalis* versus control median difference: 4.9, p < 0.001; *S agalaciae* versus control median difference: 4.5, p < 0.001; *C striatum* versus control median difference: 4.3, p < 0.001; *S mitis/oralis* versus control median difference: 4.3, p < 0.001; Fig. 3), whereas no differences were found between any species medians (all p > 0.999).

**Fig. 4:**
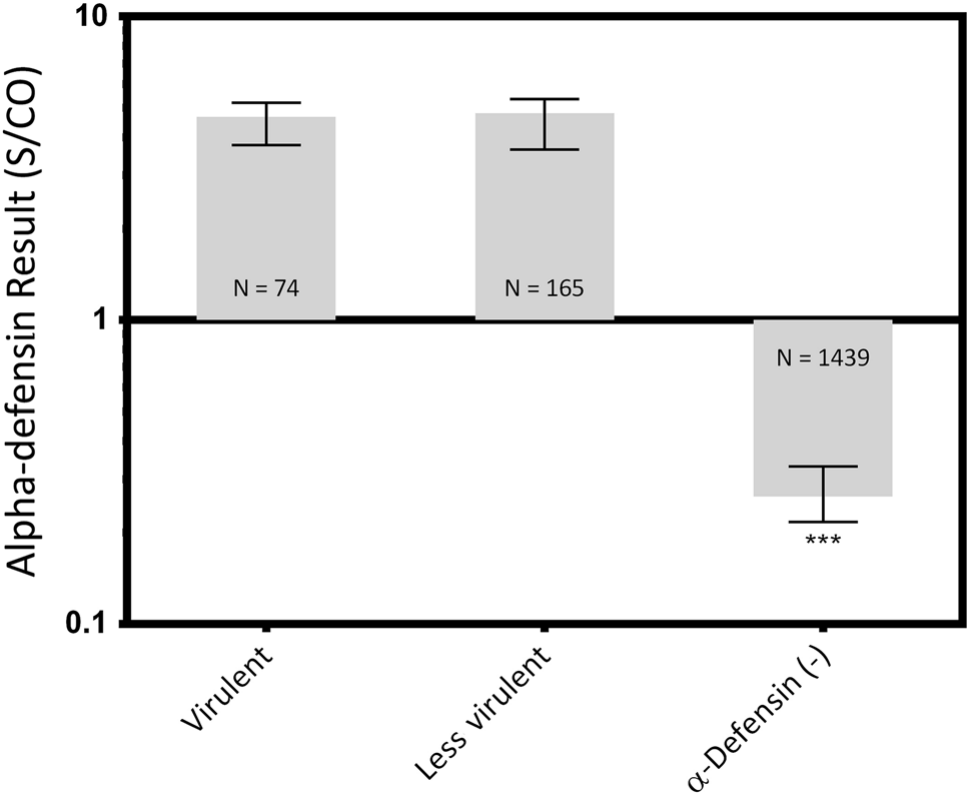
Synovial fluid samples with both a positive alpha-defensin result and a positive culture were grouped by organism virulence. Median values and the IQRs are plotted on a log scale. *** = Different with statistical significance.

No differences were found between virulent and less virulent organisms (virulent median, 4.7 [IQR, 3.8–5.2], less virulent median: 4.8 [IQR, 3.6–5.4], median difference: 0.12, p > 0.999), but both of their levels were greater than the negative control (control median: 0.26 [IQR, 0.22–0.33]; virulent versus control median difference: 4.4, p < 0.001; less virulent versus control median difference: 4.5, p < 0.001; Fig. 4).

**Fig. 5:**
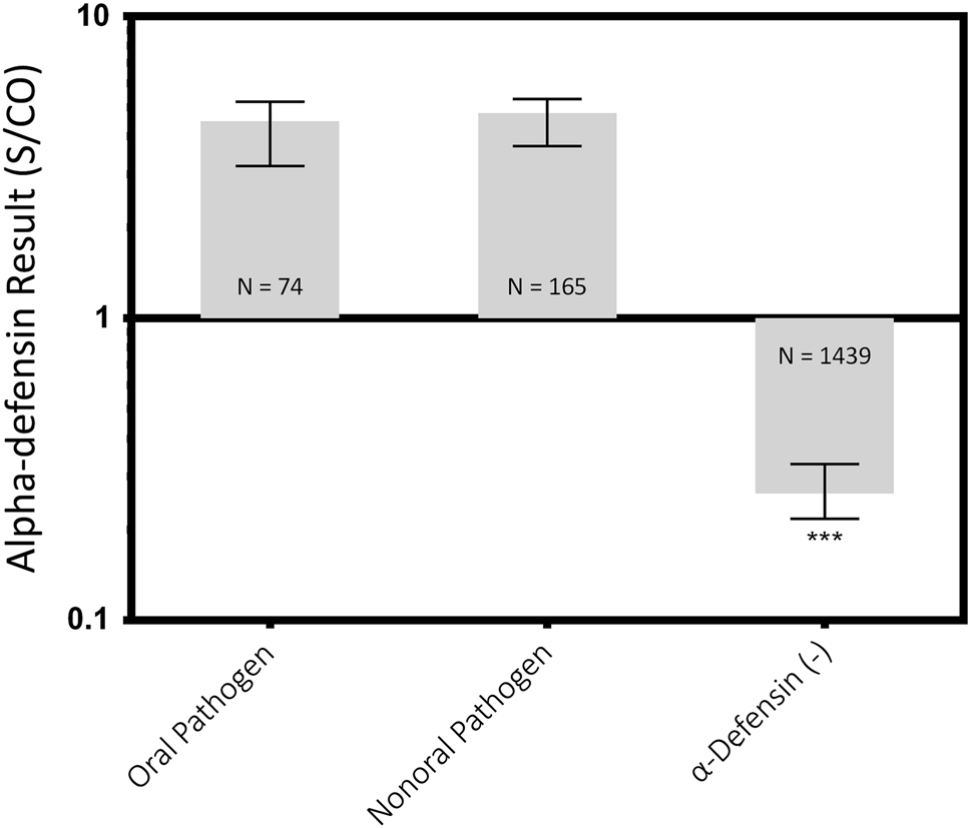
Synovial fluid samples with both a positive alpha-defensin result and a positive culture were grouped by oral pathogenicity. Median values and the IQRs are plotted on a log scale. *** = Different with statistical significance.

Similarly, no differences were found between oral and nonoral organisms (oral median, 4.5 [IQR, 3.8–5.2], nonoral median: 4.8 [IQR, 3.7–5.3], mean difference: 0.27, p > 0.999), but both of their levels were greater than the negative control (control median: 0.26 [IQR, 0.22–0.33]; oral versus control median difference: 4.2, p < 0.001; nonoral versus control median difference: 4.5, p < 0.001; Fig. 5).

**Fig. 6:**
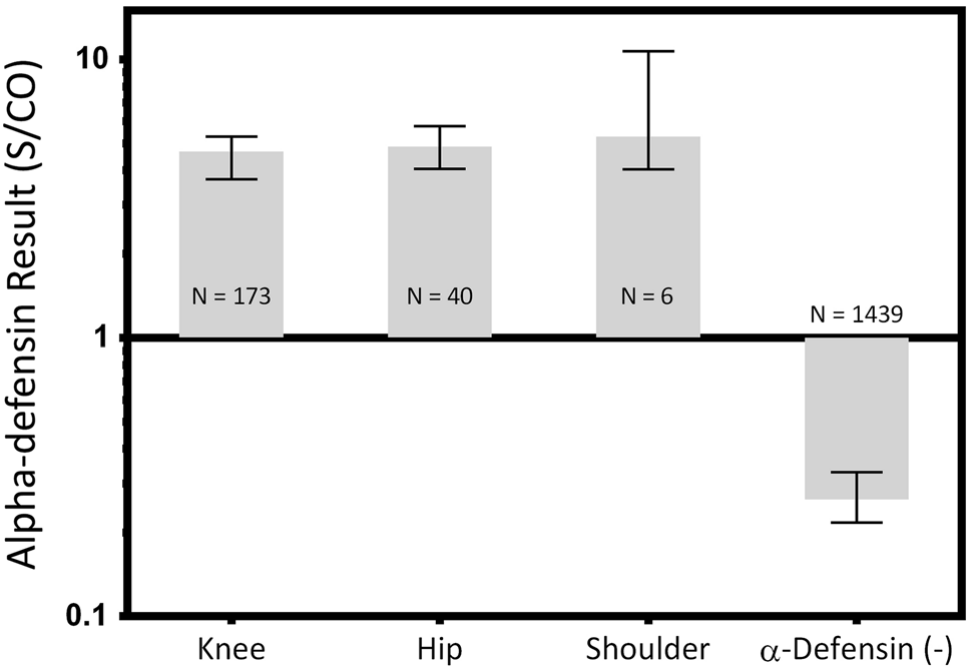
Synovial fluid samples with both a positive alpha-defensin result and a positive culture were grouped by the anatomic source. Median values and the IQRs are plotted on a log scale. *** = Different with statistical significance.

With regard to alpha-defensin levels, all anatomic sites of synovial fluid aspiration were different than the negative controls (knee median 4.7 [IQR, 3.7–5.3], hip median 4.9 [IQR, 4.1–5.8], shoulder median 5.3 [IQR, 4.0–10.7]; knee versus control median difference 4.4, p < 0.001; hip versus control median difference 4.6, p < 0.001; shoulder versus control difference, p < 0.001; Fig. 6). No differences were found between anatomic site group medians (all p > 0.999).

Overall, the median alpha-defensin level (S/CO) for all 244 alpha-defensin-positive, culture-positive samples was 4.7 (IQR, 3.7–5.3). The median alpha-defensin level (S/CO) for all 1439 alpha-defensin-negative samples was 0.26 (IQR, 0.22–0.33). The difference of the medians was 4.4 (95% confidence interval, 4.3–4.5; p < 0.001).

## Discussion

The alpha-defensin test for PJI has been demonstrated to be an accurate proxy for the MSIS definition of PJI [2, 5–7]. The most attractive aspect of this biomarker immunoassay is that it is simple, provides a final result before surgical decisions are made, and is standardized and validated to provide equivalent results for all surgeons. Although there has been great interest in the alpha-defensin test for PJI in the recent literature [4–7, 15], it is reasonable to question whether the alpha-defensin peptide is stimulated to the same extent by all organisms. On one hand, it may be desirable to have a differential alpha-defensin response to differing organisms, because organism identification may be possible. On the other hand, a differential alpha-defensin response to different organisms may be undesirable, because the accuracy of the test in diagnosing PJI may then vary by organism. The purpose of this study is to describe the breadth of organisms that can trigger a positive synovial fluid alpha-defensin test result in the setting of PJI and also to assess the magnitude of the alpha-defensin result in terms of various pathogen characteristics.

The main shortcoming of this study is the absence of a full clinical data set for every synovial fluid sample, preventing the assessment of the MSIS definition for PJI. Although these clinical data would have been ideal, the logistic and regulatory considerations in this large and geographically diverse study made such data collection unfeasible. Tissue culture data would have been especially useful for the purposes of this study, because a larger number of “culture-positive” cases would have been identified. However, this limitation should have a negligible result on this study, which did not attempt to assess alpha-defensin accuracy, but instead focused on organism-specific analysis. Several previous studies from two different clinical centers [2, 5–7] have demonstrated a consistent sensitivity and specificity ≥ 95% for the alpha-defensin test. These studies did not exclude patients on antibiotics or with comorbidities. The collection, handling, and testing of samples in this study were identical to one of these previous studies [2]. It is therefore reasonable to expect that the sensitivity and specificity of the alpha-defensin test in this study should approximate these previous publications that included substantial clinical data. Assuming a 96% specificity in this study, there should be approximately 64 false-positive results among the 498 alpha-defensin positive samples. These false-positive results would have no effect on our comparison of differing organism groups (because they would be presumably culture-negative) and would have a negligible effect on the median of the over 1400 defensin-negative controls. Assuming a 96% sensitivity in this study, there should be 17 false-negatives among the 1439 alpha-defensin-negative samples. These false-negatives would have a negligible effect on the median of the defensing-negative group and would have minimal effect on the results of this study unless all of the false-negative samples clustered into a specific organism grouping. Thus far, there is no evidence that any specific grouping of organisms is associated with false-negative alpha-defensin results. Therefore, the false-positive and false-negative alpha-defensin results in this study should have a limited effect on the results and conclusions of this study. It is worth noting that the 49% rate of positive synovial fluid cultures among alpha-defensin-positive samples in this study is quite similar to other observed rates of synovial fluid culture positivity in the setting of PJI [8, 14]. Furthermore, the 1.3% rate of positive cultures among alpha-defensin-negative fluid samples in this study is quite acceptable and actually lower than the false-positive rate of most PJI studies in the literature [8, 10, 16]. It is possible that knowledge of the organism in “culture-negative” cases could have resulted in an organism influence on the alpha-defensin levels. Some have suggested that technologies such as polymerase chain reaction (PCR) should be used to confirm results in studies such as this. However, the literature on PCR quite clearly demonstrates that it cannot be used as a gold standard and provides a generally low sensitivity with quite varied results based on technique [1, 8, 10, 11, 13].

The results of this study indicate that the alpha-defensin test for PJI is triggered by a wide spectrum of organisms with a distribution that mirrors that of the recently published literature on PJI [3, 17]. Furthermore, the alpha-defensin test appears to provide consistent results regardless of organism characteristics. When the results of this study are considered together with the results of previous studies on alpha-defensin testing [2, 5–7], the role of alpha-defensin testing in the workup of PJI becomes more evident. Currently, surgeons use many tests in combination to diagnose PJI. It is our observation that surgeons place great weight on preoperative synovial fluid culture results despite their poor sensitivity [8, 14]. Although no test is perfect, the alpha-defensin test has demonstrated the best accuracy of any individual test for PJI and also closely matches the MSIS criteria, which diagnoses PJI based on multiple laboratory results. Therefore, if the alpha-defensin result is not concordant with the presumptive diagnosis, serious consideration should be given to the possibility that the presumptive diagnosis is incorrect, triggering further clinical evaluation. This is especially true when a positive alpha-defensin test is observed in the setting of a negative synovial fluid culture, because the diagnosis of a culture-negative PJI is quite possible. As is always the case in medicine, it is very difficult to know when a new test should replace traditionally used tests. At the very least, it seems reasonable to suggest that the low-cost and accurate alpha-defensin test could replace more high-cost secondary tests for PJI that have questionable use such as bone scan and positron emission tomography. In our opinion, considering the low cost and best-in-category accuracy of the alpha-defensin test, serious consideration should be given to including the test as a standard tool for diagnosing PJI whenever synovial fluid is aspirated for a PJI workup. The alpha-defensin test may prove especially useful at institutions where a rigorous diagnostic strategy such as that described by the MSIS criteria [12] is not regularly followed.

Future research should focus on specific clinical scenarios and improved bacterial identification techniques. For example, the alpha-defensin test accuracy has yet to be tested on a large number of immunocompromised or immediate postoperative patients, although the available data suggest that the test remains accurate. Furthermore, use of the alpha-defensin test will likely result in more facile identification of culture-negative infections. Improved techniques of organism detection and identification would provide additional evidence for the diagnosis in these cases and also provide guidance for antibiotic treatment.
